# The Effect of Interfacial Chemical Bonding in TiO_2_-SiO_2_ Composites on Their Photocatalytic NOx Abatement Performance

**DOI:** 10.3791/56070

**Published:** 2017-07-04

**Authors:** Amer Hakki, Lu Yang, Fazhou Wang, Donald E. Macphee

**Affiliations:** ^1^Department of Chemistry, University of Aberdeen; ^2^State Key Laboratory of Silicate Materials for Architectures, Wuhan University of Technology

**Keywords:** Chemistry, Issue 125, Supported TiO_2_, Photocatalysis, NOx, Nitrate selectivity, environmental pollution, air quality

## Abstract

The chemical bonding of particulate photocatalysts to supporting material surfaces is of great importance in engineering more efficient and practical photocatalytic structures. However, the influence of such chemical bonding on the optical and surface properties of the photocatalyst and thus its photocatalytic activity/reaction selectivity behavior has not been systematically studied. In this investigation, TiO_2_ has been supported on the surface of SiO_2_ by means of two different methods: (i) by the *in situ* formation of TiO_2_ in the presence of sand quartz *via *a sol-gel method employing tetrabutyl orthotitanium (TBOT); and (ii) by binding the commercial TiO_2_ powder to quartz on a surface silica gel layer formed from the reaction of quartz with tetraethylorthosilicate (TEOS). For comparison, TiO_2_ nanoparticles were also deposited on the surfaces of a more reactive SiO_2_ prepared by a hydrolysis-controlled sol-gel technique as well as through a sol-gel route from TiO_2_ and SiO_2_ precursors. The combination of TiO_2_ and SiO_2_, through interfacial Ti-O-Si bonds, was confirmed by FTIR spectroscopy and the photocatalytic activities of the obtained composites were tested for photocatalytic degradation of NO according to the ISO standard method (ISO 22197−1). The electron microscope images of the obtained materials showed that variable photocatalyst coverage of the support surface can successfully be achieved but the photocatalytic activity towards NO removal was found to be affected by the preparation method and the nitrate selectivity is adversely affected by Ti-O-Si bonding.

**Figure:**
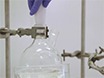


## Introduction

Concrete structures are ubiquitous in our society. Typically associated with our urban centers, their significant surface area represents an important interface with the urban atmosphere 1,2. With increasing concerns over the economic and health impacts of deteriorating urban air quality this interface presents an important opportunity for atmospheric remediation. TiO_2_-based photocatalysts have been utilized for some time in the remediation of NOx-contaminated air, and their support on these high surface area concrete structures offers concrete the additional functionality previously associated with photocatalytic materials: (i) easy-cleaning, whereby materials which bind dirt to surfaces are photocatalytically degraded enabling dirt to readily wash off with rain water 3; (ii) photo-induced hydrophilicity, which also enhances the self-cleaning effect 3; and (iii) purification of the urban atmosphere which today, is typically polluted by vehicle emissions at levels that significantly exceed maximum permissible levels, particularly with respect to NOx^4^. TiO_2_ is the most commonly employed photocatalyst in environmental applications due to its chemical stability, relatively low price, high photocatalytic activity, and more importantly its eco-safety as indicated by currently available TiO_2_ toxicology data 5.

Photocatalytic concretes have already demonstrated their potential for atmospheric remediation on trial sites throughout Europe and elsewhere. Numerous studies on photocatalytic cementitious materials over the last two decades have predominantly dealt with catalyst activity, often expressed in terms of NOx concentration reduction^1^,^6^,^7^,^8^,^9^. However, activity alone is an insufficient indicator of photocatalytic effectiveness. A reduction in NOx concentration, defined as the sum of the concentrations of the atmospheric nitrogen oxides, does not by itself represent a useful impact on air quality because the relative toxicities of the constituent gases are not equivalent 10.

Photocatalytic oxidation of NOx gases follow the sequence

NO → HONO → NO_2_ → HONO_2_ (NO_3_^-^)

The higher toxicity of NO_2_ relative to NO (by, conservatively a factor of 3^10^), means that the oxidative conversion of NO through to nitrate (*i.e.* the *nitrate selectivity*) must be maximized. Consequently, the means to deliver both high activities and high nitrate selectivities must be targeted.

As for catalysis in general, high surface areas are required for the adsorption of reacting molecules. Nanoparticulate TiO_2_ ensures the high specific surface area required for high photocatalytic activity provided particles are adequately dispersed^9^. However, when applied to concretes by mixing into the cement binder, agglomeration can occur, reducing effective surface area, and cement hydration reactions can lead to photocatalyst occlusion, reducing accessible surface area further and blocking the catalyst from activating sunlight^1^,^11^.

Significantly improved performance can therefore be expected when accessible catalyst surface area is better preserved in more efficient photocatalytic structures. These have included catalysts supported on concrete surface exposed aggregates and in zeolite structures^2^,^12^. The durability of these structures depends very much on how well bound the catalyst is to the various supports. The benefits of chemically bonding TiO_2_ to substrates have often been referred to in the literature^8^,^13^ but the means of characterizing the degree of binding has been ambiguous. Nevertheless, the integrity of a chemical bond relative to a physical attraction presents an opportunity to develop robust structures on the surface of the concrete. However, the influence of a chemical bond between TiO_2_ and a substrate,* e.g.* quartz, to provide a Ti-O-Si linkage, on the optical and photocatalytic properties of the supported TiO_2_ has not previously been studied. Therefore, the focus of the present work has been in establishing means to generate and quantify levels of Ti-O-Si linkages and to correlate these with the photocatalytic properties of the supported TiO_2_. For this purpose, commercial as well as synthesized TiO_2_ have been bonded, by different methods, onto quartz SiO_2_ sand (Q; as a simple example of an aggregate).

## Protocol

### 1. Synthesis of TiO_2_-SiO_2_ Composites

1. **Samples based on commercial quartz** NOTE: Quartz, with particles sizes in the range 20 - 100 µm was obtained by ball milling commercial quartz for 15 min and sieving. The powders were then modified with TiO_2_ by two different methods.
  1. **QT1**
    1. Prepare a 10% solution of titanium(IV) butoxide (TBOT; 97%) in ethanol as a TiO_2_ precursor^9^ by dissolving TBOT (2.6 mL) in ethanol (29.6 mL).
    2. Suspend 3 g of quartz powder in 30 g of the freshly prepared titanium precursor solution by continuous stirring.
    3. Add 0.3 mL of hydrochloric acid (32%). Stir the resulting suspension for 5 min.
    4. Add 30 mL of deionized water and continuously stir the mixture overnight.
    5. Transfer all of the viscous suspension to a Petri dish and store under ambient conditions until the solvent has completely evaporated.
    6. Wash the treated quartz with deionized water several times and then dry at 90 °C overnight.
    7. Heat-treat at 400 °C for 20 h.
    8. Cool the powders in air and sieve again to collect particles bigger than 20 µm. This was to separate modified quartz from loosely or non-connected TiO_2_.
  2. **QT2** NOTE: Support commercial photocatalyst (PC105) on quartz *via *a silica gel binder derived from tetraethyl orthosilicate (TEOS) as follows.
    1. Prepare a TEOS mother solution by dissolving TEOS (23.2 mL) in ethanol (29.2 mL). Then add deionized water (7.2 mL) and HCl (0.4 mL; 3.6 wt.%) to get final ethanol:water:HCl mixture (1:0.84:0.78 x 10^-3^ molar ratio). Stir the mixture for 10 days at room temperature.
    2. Add accurate volumes of the obtained solution to 100 mL of ethanol, in which 0.2 g TiO_2_ were suspended, to get TiO_2_:TEOS of 1:1.
    3. Stir gently at room temperature overnight and then add the suspension dropwise to 2 g of quartz with continuous stirring at 80 °C under reduced pressure.
    4. Dry the obtained powders at 90 °C overnight followed by heat treatment at 200 °C for 4 h.
2. **Samples based on synthesized silica**
  1. **ST1** NOTE: Deposit TiO_2_ nanoparticles on the surfaces of precipitated SiO_2 _prepared by a hydrolysis-controlled sol-gel technique.
    1. Synthesize monodisperse silica microspheres *via* the Stoeber-Bohn-Fink method^14^.
    2. Dissolve TEOS (5 mL) in ethanol (40 mL) and stir for 30 min (solution A).
    3. Prepare solution B by mixing ammonia solution (8 mL; 25 wt.%) with deionized water (30 mL) and ethanol (18 mL) with continuous stirring for 30 min.
    4. Quickly add all of solution A to solution B and stir at room temperature for 3 h.
    5. Collect the resulting SiO_2_ by centrifugation (1,252 x g). Wash 3 times with absolute ethanol and dry at 105 °C for 48 h.
    6. Prepare a suspension of the produced SiO_2_ by suspending 1 g in 30 mL of ethanol in an ultrasonic bath for 10 min. Stir the suspension for a further 30 min.
    7. Carefully add 1 mL of TBOT (97%) to the ethanolic SiO_2_ suspension.
    8. Age the mixture at room temperature under stirring for 24 h.
    9. Add deionized water (2 mL) and ethanol (8 mL) and then stir the mixture further for 2 h.
    10. Collect the modified powder by centrifugation and wash 3 times with ethanol. Dry at 105 °C for 48 h followed by heat treatment at 500 °C for 3 h. **NOTE: T1:** For comparison, TiO_2_ was prepared by the same method but in the absence of silica.
  2. **ST2** NOTE: In these samples, synthesize homogeneous gel of SiO_2_/TiO_2_ molar ratio 0.25 from stoichiometric mixtures of tetraethyl orthosilicate (TEOS) and titanium tetraisopropoxide (TTIP) as precursors for Si and Ti, respectively as follows.
    1. Add the required amount of TEOS (0.89 mL) dropwise into an ethanol:water:HCl mixture (73.6 mL; 1:0.84:0.78 x 10^-3^ molar ratio).
    2. Stir at room temperature for 1 h.
    3. Add the desired amount of TTIP (4.74 mL) and stir the mixture further at room temperature overnight.
    4. Achieve sol-gel conversion by stirring at 80 °C for 1 h.
    5. Heat treat the obtained gel as follows: overnight drying at 90 °C, 450 °C for 5 h, and 500 °C for 5 h. **NOTE: T2**: Pure TiO_2_ was also prepared by the same sol-gel method but in the absence of TEOS.

### 2. Characterization

1. Record IR spectra using a spectrophotometer equipped with UATR (Single Reflection Diamond)^15^.
2. Obtain X-ray diffraction (XRD) patterns using a PAN analytical diffractometer equipped with a CuKa1 1.54 Å X-ray source^16^.
3. Analyze the morphology of the samples via scanning electron microscopy (SEM), equipped with ED X-ray analyzer and BSE detector with operating voltage between 10 - 20 kV. Use energy dispersive X-ray analysis and capture images with a Digital Image Acquisition System.
4. Perform transmission electron microscopy (TEM) on a microscope operated with an accelerating voltage of 200 kV. Capture images with a camera.
5. Record UV-Vis diffuse reflectance spectra of the samples using a UV-Vis spectrophotometer equipped with fiber optic coupler. Use barium sulphate as reference in the range of 250 to 600 nm. Transform the resulting reflectance spectra into apparent absorption spectra by using the Kubelka−Munk function *F (R∞) = (1 − R∞)^2^/2R∞*^17^.

### 3. Photocatalytic Performance Test

1. Test the photocatalytic activities of the prepared materials using the removal of NOx from polluted air test^18^.
  1. For this purpose, establish an air-purification test set-up (see Figure 1) consisting of gas supplies, humidifier (2), gas flow controllers (1), photocatalytic reactor (3), UV(A) light source (4) and NOx analyzer (5). The gas supplies were NO (100 ppm) in N_2_, and synthetic air( BOC).
  2. Use mass flow controllers (1) to provide NO at 1 ppmv (0.5 ppmv, for ST1 and T1 samples) and the relative humidity to ca. 40%, confirmed by Rotronic hygropalm, to the laminar flow reactor (3) at a volume flow of 5 x 10^-5^ m^3^*s^-1^ (1.675 x 10^-5^ m^3^*s^-1^ in the case of ST1 and T1 samples).
  3. Construct the photoreactor from PMMA (Poly(methyl methacrylate)) and cover by borosilicate glass. Position it below the output from an SS0.5 kW, 500 W fully reflective solar simulator equipped with a 1.5 AM filter to ensure that the test sample (6) received a light intensity of 10 Wm^-2^ at λ <420 nm, as measured by a broadband thermopile detector.
  4. Monitor the concentrations of NO, NO_2_ and total NOx in the outlet gas flow using a NO-NO_2_-NOx Analyzer.

**Figure:**
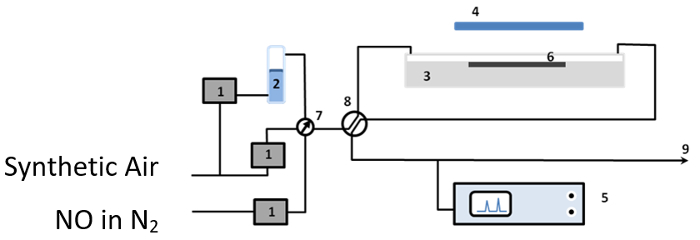

**Figure 1:The Experimental Set-up Used for the Photocatalytic Tests: (1)** mass flow controllers **(2) **humidifier **(3) **photocatalytic reactor **(4) **UV(A) light source **(5) **NOx analyzer **(6)** test sample **(7) **and** (8)** valves, and **(9)** gas stream outlet. Please click here to view a larger version of this figure.

1. Prepare test samples by pressing 0.8 g (0.3 g in the case of ST1 and T1 samples) of the material into a rectangular PMMA holder (height 0.2 cm, width 3 cm, and length 8 cm).
  1. Irradiate the resulting briquettes with a geometric surface area of 2.4 × 10^−3^ m^2^ overnight with UV (320 nm) to remove any organic contaminants adsorbed on their surfaces.

## Representative Results

*X-ray diffraction (XRD)* XRD patterns of uncoated quartz sand (Q), the prepared TiO_2_-SiO_2_ composites and TiO_2_ in the absence of quartz are shown in Figure 2. The peak positions confirm the presence of anatase in the TiO_2_ only sample as well as in the TiO_2_-SiO_2_ composites, except for the preparation at 400 °C (QT1) where no clear TiO_2_ peaks are observed. In the other cases, the differences between the different samples in peak intensities and widths are due to the differences in particle sizes and degree of crystallinity. For QT1, the lack of TiO_2_ peaks may be attributed to either a low degree of crystallinity or to a very low amount of TiO_2_ loaded on quartz under these preparation conditions. However, transmission electron microscopy (Figure 3) shows QT1 to be decorated with nanoparticulate TiO_2_ particles, which, under high magnification, are shown to be agglomerated nanospheres.

**Figure:**
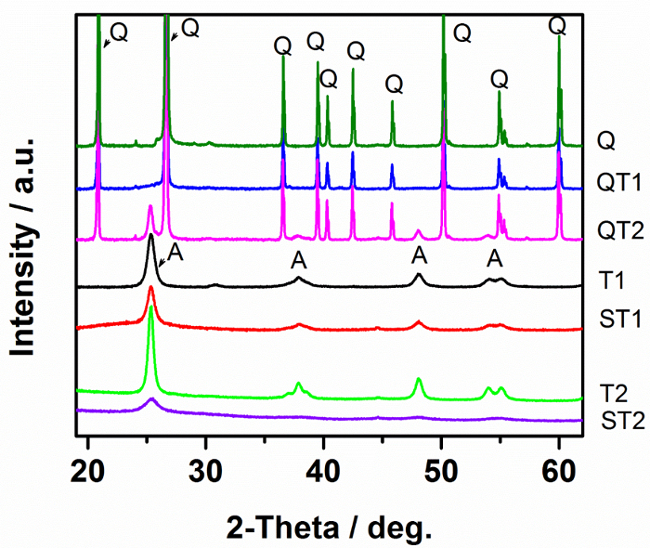

**Figure 2:** **XRD Patterns of Pure TiO_2_ and TiO_2_-SiO_2_ Composites Prepared by Different Methods.** Please click here to view a larger version of this figure.

**Figure:**
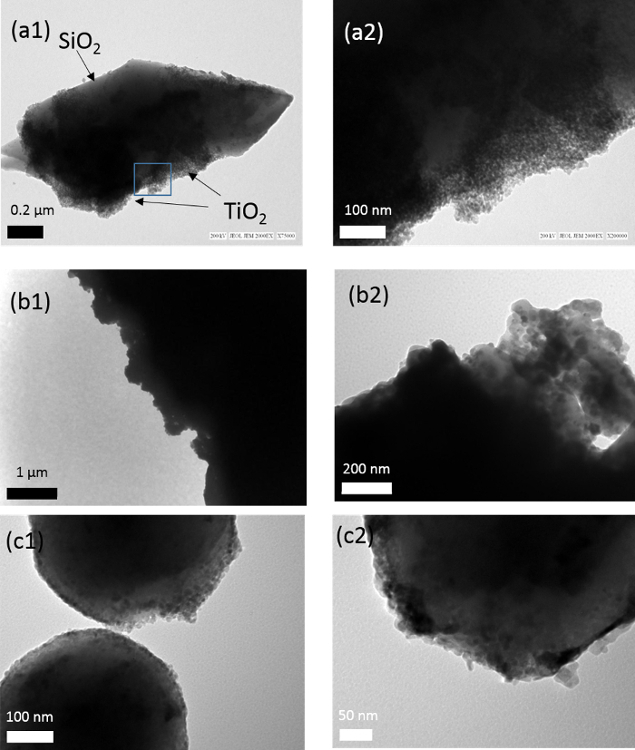

**Figure 3:TEM Images of the Titania-coated SiO_2_ Samples;(a) **QT1 **(b)** QT2 and **(c) **ST1 in low **(1) **and high **(2)** magnification images. Please click here to view a larger version of this figure.

*Diffuse reflectance spectroscopy*Figure 4 shows the UV-vis absorption spectra of the prepared samples, expressed as the modified Kubelka-Munk function [F(R_∞_)hν]^1/2^, plotted as a function of incident photon energy as required for an indirect semiconductor. The spectra are also consistent with the presence of TiO_2_ and show that TiO_2_ loading on the surface of SiO_2_ has negligible effect on the band gap. However, a small shift to higher energy level (ca. 3.3 eV) is observed for the mixed oxides sample (ST2) indicating a band gap widening effect.

**Figure:**
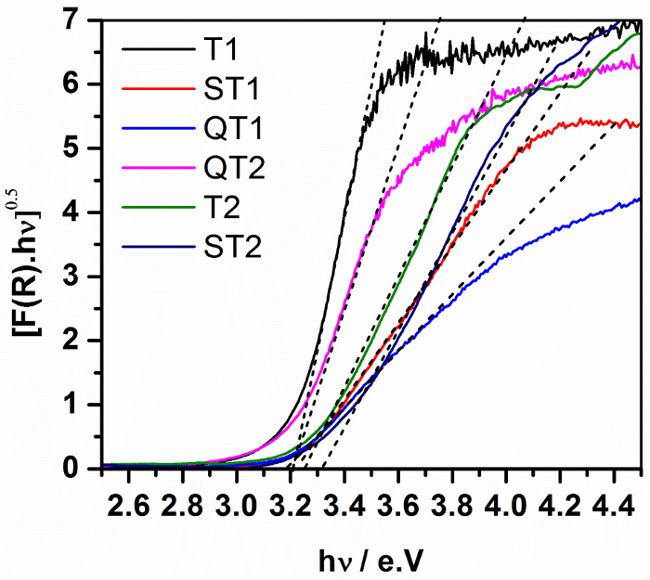

**Figure 4:** **Transformed Diffuse Reflectance Spectra (Tauc plots) for TiO_2_ and TiO_2_-SiO_2_ Composites.** Please click here to view a larger version of this figure.

*Fourier Transform Infrared Spectroscopy (FTIR)*Figure 5 shows the FTIR spectra of the SiO_2_/TiO_2_ mixed oxides samples and of the TiO_2_-Q composites. Evidence for the chemical binding of TiO_2_ to SiO_2_ may be observed in the range between 900 - 960 cm^-1^ assignable to the Si-O-Ti stretching vibrational mode^15^; as expected, no absorption peak due to this mode was observed for SiO_2_ or TiO_2_.

**Figure:**
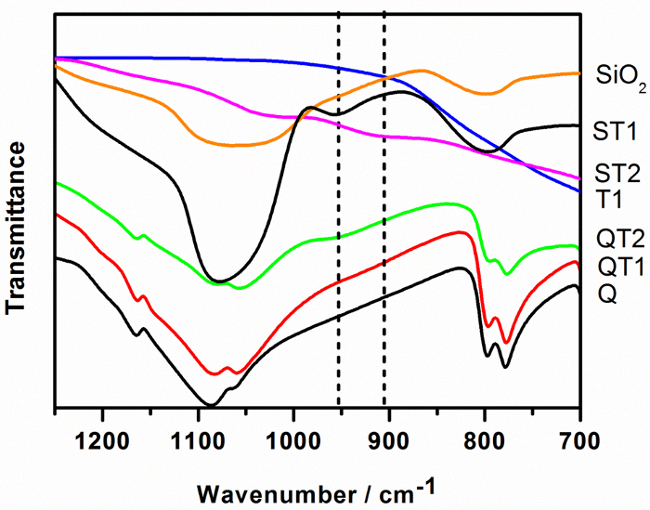

**Figure 5:FTIR Spectra of TiO_2_ (T1), SiO_2_, Reactive Silica Modified with TiO_2_ (ST1), SiO_2_-TiO_2_ Mixed Oxide Prepared *via *Sol-gel Method (ST2), Quartz (Q) and TiO_2_-modified Quartz Samples (QT1, QT2)**. For clarity, the spectrum for T2 is not shown but it is identical to T1. Please click here to view a larger version of this figure.

*TiO_2_-quartz composites* Although molecular modeling by Tokarsky *et al.*^16^ indicated the possibility of Ti-O-Si on sand quartz, they were not able to observe clear evidence for Ti-O-Si experimentally following thermal hydrolysis of titanyl sulphate in the presence of quartz. However, as can be seen from Figure 5, a very low IR absorption can be noticed in the range 920 - 960 cm^-1^ for comparable QT1 composites in this study indicating a small amount of Ti-O-Si bonding. QT2 exhibits more significant absorbance likely to be associated with the interaction of TiO_2_ with more reactive TEOS coating the quartz surface. It is likely that the TiO_2_ is associated with the resulting silicate-based gel rather than the quartz surface.

*Mixed oxide systems* The highest FTIR absorption measured in this study was observed for ST2, derived from the reaction of organic precursors. Such a system is expected to maximize the dispersion and mixing of reactants which is consistent with the FTIR data. ST1 utilized a pre-precipitated silica but despite its reactive surface, the resulting FTIR absorption indicates a relatively low level of bonding.

*Scanning electron microsocopy (SEM) * The effectiveness of a silicate-based film on quartz (QT2) for the efficient support of TiO_2_ has been examined by SEM. Much depends on how well the film itself coats the quartz substrate. Figure 6 compares the SEM-EDS of commercial TiO_2_ (PC105) dispersed within this film derived from TEOS with TiO_2_ in a 1:1 molar ratio (QT2). The silicate film was found to have been immobilized inhomogenously on the grains as some areas remain clear of the silicate coating. Consequently, in this case, TiO_2_, associated with the silicate-based gel phase, is also inhomogeneously distributed and is not bonded directly to the quartz surface. This is consistent with the TEM image in Figure 3 b(2). The silicate coating (top right of image) gives an EDS analyses comparable with that reported in Figure 6(d) indicating the association of TiO_2_ with the silicate layer.

**Figure:**
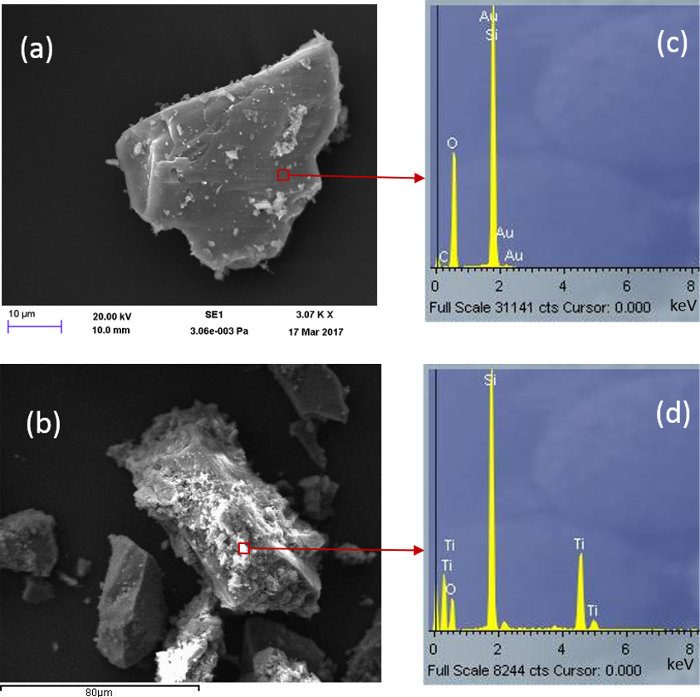

**Figure 6:SEM Images for (a) Bare Quartz and (b) Sample QT2. **Corresponding EDS data are presented in **c** and **d** respectively. Please click here to view a larger version of this figure.

### Photocatalytic performance

Figure 7 shows an example of the changes in the concentrations of NO, NOx, and NO_2_ in the gas stream flows over TiO_2_ (PC105) in the dark and under illumination. When light was switched on, the initial NO concentration drops by ca. 48% with a simultaneous formation of NO_2_. Consequently, the concentration of NOx, which expresses mainly the sum of the NO and NO_2_ concentrations, is reduced during illumination time.

The proposed conversion pathway of NO after its adsorption on illuminated TiO_2_-based photocatalyst can be summarized in the following scheme:

NO → HONO → NO_2_ → HONO_2_ (NO_3_^-^)

It can also be noticed from Figure 7 that the concentration of NO increased slightly and continuously during the entire irradiation time. This illustrates an approach to the steady state condition and can be attributed to the accumulation on the available active sites of photocatalytically generated NO oxidation products, *i.e.*, HNO_2_/NO_2_^-^; NO_2_; and HNO_3_/NO_3_^-^, which may influence NO adsorption rates. Bloh et al. reported that achieving a steady-state in this system requires several hours of illumination.

**Figure:**
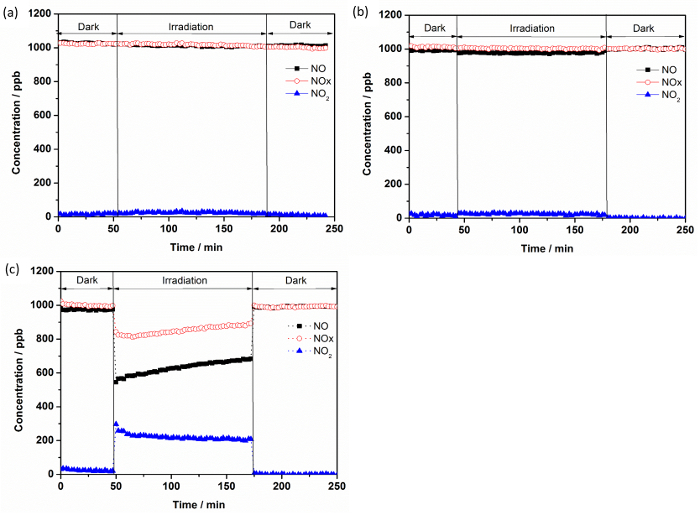

**Figure 7: Concentration Variations for NO, NO_2_, and NOx as a Function of Time:(a)** without any photocatalyst or support **(b)** quartz only, and **(c) **PC105. Please click here to view a larger version of this figure.

To determine and compare the activities of the obtained TiO_2_-SiO_2_ composites for NOx abatement, the photonic efficiencies (ξ) for the removal of NO, NOx and the formation of NO_2_ was calculated and illustrated in Figure 8.

**Figure:**
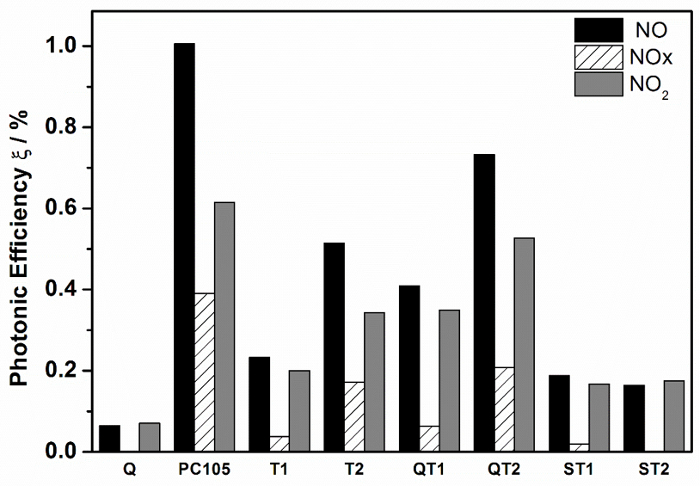

**Figure 8:Photonic Efficiencies of Different TiO_2_ and TiO_2_-SiO_2_ Composite Powders for NO and NOx Removal and for NO_2_ Formation.** Directly comparable systems are identified with the same symbols, supported *vs* non-supported pairs. Please click here to view a larger version of this figure.

ξ is defined as the ratio of the reaction rate and the incident photon flux and was calculated according to Eq.(9)^18^, where 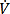 is the volumetric flow rate; c_d_ the concentration of NO, NOx, or NO_2_ under dark conditions; c_i_ the concentration of the same gas under illumination; p the pressure; N_A_ the Avogadro constant; h is the Plank constant; c is the speed of light; I the incident irradiation intensity, λ the employed wavelength assuming monochromatic light (365 nm), A the irradiated area; R the gas constant; and T the absolute temperature.


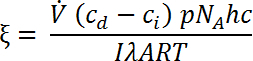
 (9)

## Discussion

Figure 8 shows quite significant differences between the NO photonic efficiencies for each of the photocatalytic materials. The advantages of supporting the photocatalyst to increase accessibility to reactive surface is now well established and it is worth noting the difference between the photonic efficiencies for NO oxidation measured for PC105 and for PC105 supported on treated quartz (QT2). ξ NO (QT2) was measured at 73% of that for PC105 but QT2 had only 6.5% of the TiO_2_ loading. Clearly, activity improvements are significant on supported systems but care should be applied when interpreting measurements with significant morphological differences.

A key characteristic of the photocatalytic test system which can be expected to influence the measurement is the surface texture of the sample supported in the photocatalyst reactor. This influences the effective surface area. The calculation of ξ includes an area term but this is a two-dimensional area of illumination defined by the reactor sample holder. The particle size distribution of TiO_2_ powders, i.e. PC105, T1 and T2, are quite different from the composites, where TiO_2_ 'powder' is supported on SiO_2_ of diameter in the range 0.4-50 μm. This means that the photocatalyst surface textures are quite variable and are expected to influence the reported photonic efficiencies. It also influences reactor flow characteristics. The rougher the texture, due to packing characteristics, the more likely that the laminar flow regime required is disrupted. This is expected to influence rates of gas molecule diffusion to surface and consequently the photonic efficiency measurement.

As a consequence of these effects, the most useful comparison of photocatalyst types must be based on properties derived from measurements on individual catalysts. In this study, nitrate selectivity, which is based on ξ NO and ξ NO_2_ (Equation 10), both measured on the same sample are used in subsequent discussion.


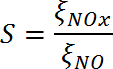
(10)

**Figure:**
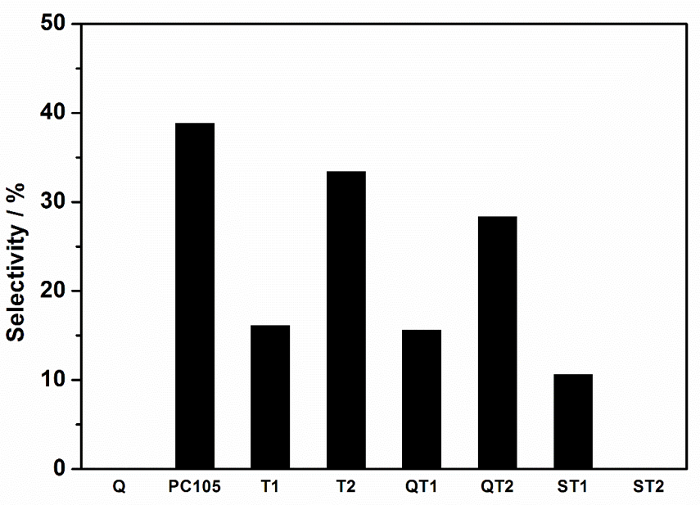

**Figure 9: Selectivity Towards the Total Removal of NOx,* i.e.*, Nitrate Selectivity, Recorded for Different TiO_2_ and TiO_2_-SiO_2_ Composite Powders.** Directly comparable systems are identified with the same symbols, supported *vs* non-supported pairs. Please click here to view a larger version of this figure.

The factors which control nitrate selectivity appear to be complex and relevant variables include TiO_2_ polymorphism, defect state, availability of water, *etc.*^7^, but the role of substrate binding, often considered to be advantageous to photocatalytic performance, can now also be considered. It is beneficial therefore to discuss the nitrate selectivity differences between non-bonded and bonded systems, i.e. stand-alone photocatalyst *vs* photocatalyst-support composites, *e.g.* PC105 *vs* QT2; where QT2 represents PC105 supported in a silicate coating on quartz. These nitrate selectivity differences are summarized in **Table 1**.

**Table:** 

**Photocatalyst**	**Photocatalyst-support**	**DSelectivity (%); (relative selectivity reduction (%))**	**FTIR peak area ratio; (Ti-O-Si) /SiO2**	**Ti-O-Si peak centre (cm^-1^)**
PC105	QT2	(38.8-28.3) = 10.5; (-27)	0.0088	960
T1	ST1	(16.0-10.6) = 5.4; (-34)	0.0184	960
T2	ST2	(33.4-0) = 33.4; (-100)	0.6566	920
T1	QT1	(16.0-15.6) = 0.4; (-3)	0.0014	930

**Table 1: Influence of Composite Formation and Ti-O-Si Bonding on Photocatalyst Performance. **Background corrected FTIR peak areas for peaks assigned to Ti-O-Si (920 - 960 cm^-1^) and for SiO_2_ (990 - 1230 cm^-1^) were obtained from Figure 5 using Origin Peak Analyses software. The dimensionless area ratio indicated in **Table 1** is taken as a measure of the degree of Ti-O-Si bonding in composite systems. Also shown are the peak center positions associated with the Ti-O-Si linkage. These data are summarized in Figure 10.

**Figure:**
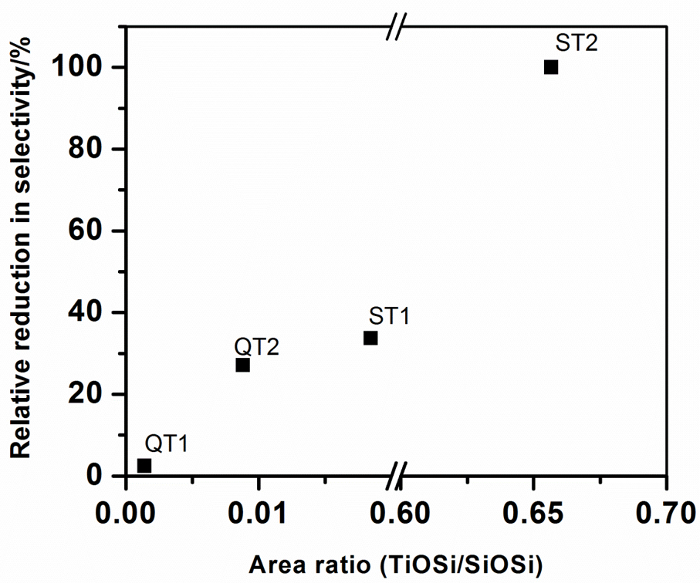

**Figure 10:** **Relative Reduction in Nitrate Selectivity for Different TiO_2_ Materials Combined with SiO_2_ as a Function of Ti-O-Si Bonding in Photocatalyst-support Composites.** Please click here to view a larger version of this figure.

The greatest selectivity reduction on composite formation,* i.e.* that which would show the largest negative impact on ambient air quality, is indicated for photocatalyst T2 when it is combined with a silicate precursor. A highly dispersed gel is produced in which Ti-O-Si linkages are maximized. The peak area analyses indicates that around 65 mole % of the TiO_2_ is associated with SiO_2_ through Ti-O-Si connections, which is approaching the stoichiometric TiO_2_:SiO_2_ ratio of the preparation (80%) and providing confidence in the peak area ratio analysis. It is also noteworthy that the Ti-O-Si peak center is located at the lowest wavenumber observed for the composites and suggests that compositional information may be embedded in the Ti-O-Si peak characteristics. All other composites display considerably lower (Ti-O-Si)/SiO_2 _peak area ratios, indicating lower levels of Ti-O-Si bonding. Figure 10 shows that this level of bonding is correlated with Selectivity, expressed as a percentage reduction from the free standing catalyst selectivity, indicating that Ti-O-Si binding has a negative impact on photocatalytic NOx abatement.

The consequences of these findings are that a compromise must be met to ensure the physical durability of a bonded system without a significant loss of photocatalytic performance. Possible approaches could include: (i) increasing the supported TiO_2_ particle size such that the beneficial Ti-O-Ti bonding, which defines the intrinsic photocatalytic properties of 'stand-alone' photocatalysts, are not diluted by the Ti-O-Si linkages, and/or (ii) engineering a thin, porous and durable surface coatings for the substrate such that the photocatalyst is trapped in pores accessible to reactant gas molecules and illumination.

Silica in the form of quartz sand or reactive silica spheres has been successfully modified with TiO_2_ either via binding commercial TiO_2_ photocatalyst (PC105), utilizing a silicate-based binder or via the hydrolysis-condensation reactions of different Ti precursors. The photocatalytic performance of the resulting composites has been compared with that of a sol-gel derived mixed oxide system promoting high levels of Ti-O-Si binding linkages. The key findings show that: (i) the degree of TiO_2_-SiO_2_ binding in the mixed oxide preparation is high (65%) as expected and approaches the stoichiometric TiO_2_:SiO_2_ ratio in the preparation. This composite gel system displayed no nitrate selectivity compared with the comparable sol-gel derived TiO_2_ (T2) which showed a selectivity of 33%, (ii) as the reactivity of the silicate surface reduces, the degree of Ti-O-Si binding reduces; the order is reactive silica spheres (ST1) > silicate gel layer on quartz (QT2) > bare quartz, and (iii) the nitrate selectivity of TiO_2_ is adversely affected by the level of Ti-O-Si bonding.

## Disclosures

Authors have nothing to disclose.
